# Effects of synbiotic supplementation on energy and macronutrients homeostasis and muscle wasting of critical care patients: study protocol and a review of previous studies

**DOI:** 10.1186/s13063-020-4136-3

**Published:** 2020-02-24

**Authors:** Najmeh Seifi, Mohammad Safarian, Mohsen Nematy, Reza Rezvani, Majid Khadem-Rezaian, Alireza Sedaghat

**Affiliations:** 1 Department of Nutrition, Medical School, Mashhad University of Medical Sciences, Mashhad, Iran; 2 Metabolic Syndrome Research Center, Mashhad University of Medical Sciences, Mashhad, Iran; 3 Department of community medicine, Medical School, Mashhad University of Medical Sciences, Mashhad, Iran; 4 Department of Anesthesiology, Mashhad University of Medical Sciences, Mashhad, Iran

**Keywords:** Synbiotics, Homeostasis, Muscle wasting, Critical care, Gut microbiota

## Abstract

**Background:**

An extreme and persistent dysbiosis occurs among critically ill patients, regardless of the heterogeneity of disease. Dysbiosis in critically ill patients may make them prone to hospital-acquired infections, sepsis, multi-organ failure (MOF), energy homeostasis disturbance, muscle wasting, and cachexia. Modulation of gut microbiota through synbiotics can be considered as a potential treatment for muscle wasting and macronutrient homeostasis disturbances.

**Methods:**

This is a prospective, single-center, double-blind, parallel randomized controlled trial with the aim to evaluate the effects of synbiotic supplementation on energy and macronutrient homeostasis and muscle wasting in critically ill patients. A total of 40 hemodynamically stable, adult, critically ill patients who receive enteral nutrition via a nasogasteric tube (NGT) in the 24–48 h after admission to critical care will be included in this study. Eligible patients will be randomly assigned to receive Lactocare (ZistTakhmir) capsules 500 mg every 12 h or a placebo capsule, which contains only the sterile maize starch and is similar to synbiotic capsules for 14 days. The synbiotic and placebo capsules will be given through the nasogastric tube, separately from gavage, after feeding.

**Discussion:**

Gut microbiota modulation through synbiotics is proposed to improve clinical prognosis and reduce infectious complications, ventilator dependency, and length of ICU stay by improving energy and macronutrient homeostasis and reducing muscle protein catabolism.

**Trial registration:**

Iranian Registry of Clinical Trials, IRCT20190227042857N1. Registered on 17 March 2019.

## Background

### Gut microbiota, dysbiosis and critical illness

The gut microbiota refers to the commensal microorganisms that reside in our gastrointestinal tract (GIT) with a symbiotic relationship [1]. Gut microbiota has a significant role in host metabolism and homeostasis [2, 3]. A disturbance in this microbial community, which leads to an unhealthy state, is called dysbiosis [4]. Over the past decade, emerging evidence has demonstrated the role of intestinal dysbiosis in the pathogenesis of various conditions, such as infectious, immune, and metabolic diseases [5], while it has not been studied extensively in critical illness. An extreme and persistent dysbiosis occurs among critically ill patients, regardless of the heterogeneity of disease. The extreme dysbiosis in patients under critical care is due to the stress of critical illness, multiple antibiotics and additional pharmacological interventions, and highly processed enteral/parenteral nutrition [6, 7]. Dysbiosis in critically ill patients may make them prone to hospital-acquired infections, sepsis, multi-organ failure (MOF), energy homeostasis disturbance, muscle wasting, and cachexia [6, 8, 9].

### Dysbiosis and energy homeostasis in critical illness

The majority of patients in the intensive care unit (ICU) have had a severe illness, trauma, or major surgery, and accordingly they are unable to manage their nutritional demands. Although nutritional support is a daily practice in the ICU, many patients still suffer from malnutrition due to lack of intake or uptake of nutrients [10]. The prevalence of malnutrition in the ICU within developed and developing countries is reported as 50.8% and 78.1%, respectively [11]. Malnutrition is independently associated with longer ICU stay, more ICU readmissions, and a higher incidence of infections and risk of mortality [11]. A greater degree of malnutrition is also associated with a higher risk of 28-day mortality [12]. Malnutrition further tends toward acute or chronic loss of muscle bulk and function [13]. The gut microbiota and their derived metabolites play an essential role in the absorption, storage, and consumption of energy derived from the diet [14, 15]. Previous studies suggest that modulating gut microbiota by novel therapeutics, such as prebiotics, probiotics, or synbiotics, can have an effect on gastrointestinal tolerance and complications of enteral nutrition, which eventually lead to the regulation of energy intake. Recently, Tuncay et al. showed that enteric formula with prebiotic content in patients under neurocritical care was associated with a significant increase in total feed volume and energy intake and a non-significant tendency to achieve a target dose of nutrition more frequently and earlier [16]. Malik et al. also found that in patients in the ICU, receiving enteral formula supplemented with probiotics led to a faster return of gut function (tolerated feeding of 80% of their estimated required calories for 48 h consecutively) [17]. However, Sanaie et al. demonstrated that daily energy and protein intake in patients receiving probiotic supplements on the ICU were not significantly different from the group receiving placebo [18]. In the critical care setting, diarrhea is the most obvious complication of enteral nutrition (EN), which is associated with the inadequacy of energy and macronutrient intake [16]. Previous systematic reviews have confirmed the significant benefit of probiotics in the reduction of diarrhea in hospital patients overall. But a recent meta-analysis focused on patients in the ICU found no benefit of probiotics in preventing or treating diarrhea [19]. Besides, in the dysbiosis state of critical illness, microbial products that reach distant organs like brain, adipose tissue, and liver, favor the development of immune-mediated diseases and metabolic alterations [20]. Gut microbiota metabolites like short-chain fatty acids (SCFAs) can also influence the immune system and host metabolism, which regulates energy homeostasis [20]. Thus, gut microbiota modulation may be beneficial in the regulation of immune and metabolic responses and energy homeostasis.

### Dysbiosis and muscle wasting in critical illness

**Fig. 1:**
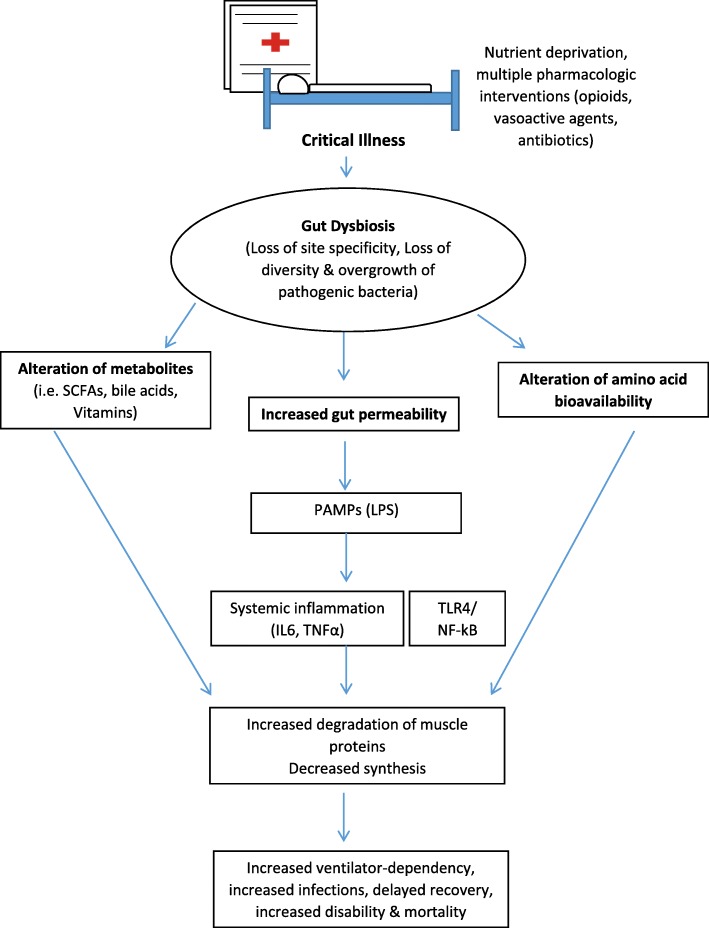
Dysbiosis and muscle wasting in critical illness. Abbreviations: EN, enteral nutrition; IL6, interleukin 6; LPS, lipopolysaccharides; NF-kB, nuclear factor kappa light chain enhancer of activated B cells; PAMPs, pathogen-associated molecular patterns; SCFAs, short-chain fatty acids; TLR4, toll-like receptor 4; TNFα, tumor necrosis factor alpha

Muscle wasting, characterized by loss of muscle mass and strength, is associated with negative health outcomes such as functional disability, greater risk of infections, delayed recovery, poor life quality, and mortality [21]. The gut microbiota have been reported to influence muscle metabolism. The molecular mechanisms of this gut-muscle axis remain to be identified. Gut microbiota influence amino acid bioavailability and are sources of different metabolites, such as conjugated linoleic acid, acetate, and bile acids, which modulate muscle metabolism [8]. Various pathogen-associated molecular patterns (PAMPs) activate the transcription factor nuclear factor kappa light chain enhancer of activated B cells (NF-kB) through toll-like receptors (TLRs) and modulate the production of proinflammatory cytokines, which can induce muscle atrophy [8] (Fig. 1).

Modulation of gut microbiota through prebiotics, probiotics, or synbiotics can be considered as a potential treatment for muscle wasting and cachexia. In mouse models of leukemia, restoring Lactobacillus species by oral supplementation with *Lactobacillus reuteri* 100–23 and Lactobacillus gasseri 311,476 reduces inflammatory cytokines and expression of muscle atrophy makers [22]. In another study, Bindels et al. showed that prebiotic supplementation in leukemic mice could contribute to delaying anorexia and fat mass sparing by inducing a metabolic shift in adipose tissue [23]. In the mouse models of cancer cachexia, administration of a synbiotic supplement including inulin-type fructans and live *L. reuteri* 100–23 was associated with restoration of the gut barrier and immune function, thus reducing cachexia. It also prolonged survival [24]. Varian et al. also showed that probiotic administration in leukemic mice could inhibit cachexia by reducing systemic inflammation [25].

### Study rationale

Considering the extreme dysbiosis in critically ill patients and related energy and macronutrients homeostasis disturbance and muscle wasting, prompted us to evaluate the effect of synbiotic supplementation on the elimination of this condition. To our knowledge, this is the first study to investiage the effect of synbiotic supplementation on muscle wasting in critically ill patients.

## Study objectives

### Primary objective

The primary objective is to evaluate the effects of synbiotic supplementation on energy and macronutrient homeostasis and muscle wasting in patients under critical care.

### Secondary objectives

The secondary objective is to evaluate the effects of synbiotic supplementation on infectious complications and length of hospital and ICU stay in patients under critical care.

## Study design

This is a prospective, single-center, double-blind, parallel randomized controlled trial that will be conducted in Edalatian Medical ICU, Emam Reza Hospital, Mashhad, Iran. The study protocol was written following the Standard Protocol Items: Recommendation for Interventional Trials (SPIRIT) Checklist (Additional file 1).

## Selection and enrollment of participants

### Inclusion criteria

Participants must meet all the inclusion criteria to participate in this study: adults aged 18–65 years; admitted to the ICU; hemodynamically stable within 24–48 h after admission; requiring exclusive enteral nutrition (EN) via a nasogastric tube (NGT); not taking any kind of microbial cell preparations (prebiotics probiotics, synbiotics); estimated length of ICU stay more than 14 days; and provision of written consent.

### Exclusion criteria

All candidates meeting any of the exclusion criteria at baseline will be excluded from study participation: pregnancy or lactation; any contraindication to EN; any contraindication to insertion of the NGT; receiving immunosuppressive treatment, radiotherapy, or chemotherapy; hematologic disease; acquired immune deficiency syndrome (AIDS); transplant recipient; known allergy to microbial cell preparations; cancer or autoimmune diseases; artificial heart valve or congenital heart valve disease; estimated length of ICU stay less than 4 days; gastric disease; or gastrointestinal tract (GI) tract surgery.

### Study enrollment procedure

Before the screening procedure, informed consent will be obtained from every participant who meets the inclusion criteria. First, we will describe the purpose of the study, the procedures involved, the length of time the subject is suspected to participate, any possible disadvantages or discomforts, the benefits of the study to society and individuals, and the person to contact for answers to further questions. We will also emphasize that participation is voluntary, and refusal or withdrawal will not cause any loss of benefits that they are entitled to receive. Then the participants or their legal guardian will read and sign two copies of the written form. If, the informed consent was obtained from the patient’s guardian because of the patient’s lack of competence to consent and then later in the study the patient subsequently became competent as required, consent will be regained.

### Random allocation and blinding

After providing their written consent, patients are randomly allocated in a 1:1 ratio to the intervention or control group (A or B). Patients will be randomized through a stratified sequential randomization plan generated online. Randomization will be stratified by disease severity (Acute Physiology and Chronic Health Evaluation (APACHE) II,^1^ 0–35 and 35–70). For allocation concealment, we will use sealed opaque envelopes; inside each there is a carbon paper and the A or B card. To avoid probable selection bias, we will write the patient’s name on the envelope before opening it. All patients, researchers, and medical staff will be blinded to the allocation to either synbiotic or placebo capsules. An available third party, the secretary of the ICU, will be aware of whether A or B is allocated the synbiotic supplement. In the case of any complication associated with the intervention allocated, the medical staff will refer to the secretary for details.

## Study interventions

### Interventions, administration, and duration

**Fig. 2:**
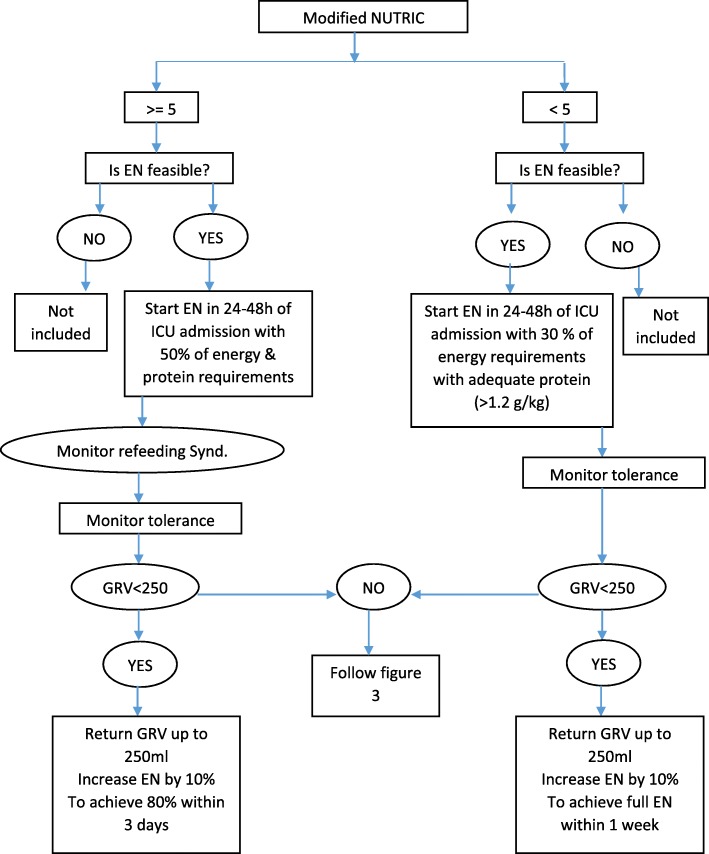
Flow chart for EN initiation and goal achievement. Abbreviations: EN, enteral nutrition; GRV, gastric residual volume; ICU, intensive care unit; ml, milliliter; NUTRIC, Nutrition Risk in Critically Ill

**Fig. 3:**
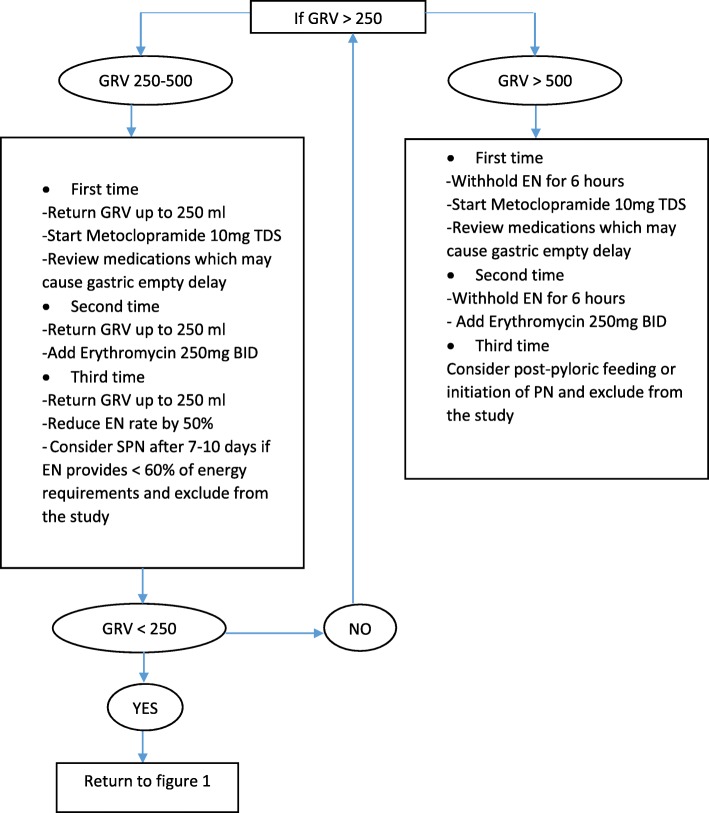
Flow chart of GRV management. Abbreviations: EN, enteral nutrition; GRV, gastric residual volume; ICU, intensive care unit; ml, milliliter; NUTRIC, Nutrition Risk in Critically Ill; SPN, supplementary parenteral nutrition

All eligible patients will receive standard hospital gavage as EN through the NGT in the 24-48 h after admission to the ICU. According to the recent European Society of Parenteral and Enteral Nutrition (ESPEN) guideline on clinical nutrition in the ICU [26], continuous rather than bolus EN is preferred because it causes less diarrhea, but there is no difference in other outcomes. Another systematic review showed that bolus feeding is associated with lower aspiration rate and better calorie attainment [27]. It also provides a greater stimulus for protein synthesis [28]. Considering these data and the availability of bolus EN in our hospitals we applied this method. In the absence of an indirect calorimeter, the simple weight-based equation of 20–25 Kcal/kg/day in the acute flow phase and 25–30 Kcal/kg/day in the anabolic flow phase is preferred for measurement of calorie requirements. For overweight and obese patients, ideal body weight: 0.9 × height (cm) − 100 (male) (or − 106 (female) is suggested as a reference weight [26]. To avoid overfeeding, the EN target will be prescribed within 3 days in patients with high nutritional risk and within 7 days in patients with low nutritional risk according to the modified Nutrition Risk in Critically Ill (NUTRIC)^2^ score. The flow charts in Figs. 2 and 3 will be used for initiation and continuation of EN.

In the intervention group, patients will receive Lactocare (ZistTakhmir) capsules 500 mg every 12 h for 14 days. Each capsule contains *Lactobacillus casei* 1.5 × 10^9^ colony-forming units (CFU), *Lactobacillus acidophilus* 1.5 × 10^10^ CFU, *Lactobacillus rhamnosus* 3.5 × 10^9^ CFU, *Lactobacillus bulgaricus* 2.5 × 10^8^ CFU, *Bifidobacterium breve* 1 × 10^10^ CFU, *Bifidobacterium longum* 5 × 10^8^ CFU, *Streptococcus thermophilus* 1.5 × 10^8^ CFU, and fructooligosaccharides (FOS). The probiotics capsule will be given through the NGT, separately from gavage, after feeding. Patients in the control group will receive a placebo capsule, which contains only sterile maize starch and is similar to probiotic capsules. The liquid preparations ready for gavage through the NGT are also similar in color and odor.

### Handling of the study intervention

The pharmaceutical company will provide synbiotic and placebo capsules in distinct boxes identified as A or B. Synbiotic capsules can be stored at room temperature for 2–3 weeks but the best condition for keeping this product is in the refrigerator at 2–8 °C. Unused study products will be returned to the company supplying them.

### Concomitant interventions

Concomitant interventions will be:

1. Antibiotics

- It is common that patients under critical care receive at least one antibiotic during their ICU stay. On the other hand, it is believed that antibiotics have bacteriostatic or bactericidal effects on both pathogenic and non-pathogenic bacteria. So, it is recommended that probiotic and antibiotic administration be separated by at least 2 h hours [29].

2. Opioids

- Considering their analgesic and sedative properties, opioids are widely used in patients under critical care. Opioids are believed to suppress the immune system and delay GI peristalsis [30]. Delayed peristalsis can increase bacterial translocation out of the GI tract [31].

3. H2 receptor blockers

- Prevention of GI tract (GIT) stress ulcers, through H2 receptor blockers or proton-pump inhibitors, is common in critical care practice. Increase in GI acidity can cause overgrowth of some pathogenic bacteria [32].

4. Catecholamines

- It is believed that the elevated level of catecholamines in patients under critical care, as prescribed exogenously beside endogenous production, can impair the immune system [33].

These drugs are routinely administered in critical care practice, so we will record and consider them as conflicting factors.

### Adherence assessment

As the researcher will administer the capsules to the patients through the NGT, adherence assessment is not required.

## Study procedures

### Schedule of evaluations

The schedule of evaluations is shown in Table 1.

**Table 1:** Schedule of enrollment, intervention, and assessments in accordance with the Standard Protocol Items: Recommendations for Interventional Trials (SPIRIT) guidelines

Study period																	
	Enrollment	Allocation	Post-allocation														Close- out
Time point	Pre- allocation	Pre- intervention	1	2	3	4	5	6	7	8	9	10	11	12	13	14	15
Enrollment																	
Informed consent form	×																
Eligibility	×																
Demographics	×																
Intervention			×	×	×	×	×	×	×	×	×	×	×	×	×	×	
Assessments																	
APACHEII, Modified NUTRIC, SOFA		×								×							×
GCS and vital signs		×	×	×	×	×	×	×	×	×	×	×	×	×	×	×	
Energy homeostasis		×	×	×	×	×	×	×	×	×	×	×	×	×	×	×	×
Abdominal examination		×	×	×	×	×	×	×	×	×	×	×	×	×	×	×	
Fluid balance examination		×	×	×	×	×	×	×	×	×	×	×	×	×	×	×	
GRV																	According to Fig. 3.
Mid-arm circumference		×	×			×			×			×			×		×
Infectious events		×	×	×	×	×	×	×	×	×	×	×	×	×	×	×	×
Pressure ulcer		×	×	×	×	×	×	×	×	×	×	×	×	×	×	×	×
Mg, P, Na, K, Cl		×	×	×	×	×	×	×	×	×	×	×	×	×	×	×	
Glucose		×	×	×	×	×	×	×	×	×	×	×	×	×	×	×	×
Insulin		×															×
AST, ALT,TG, Urea		×				×			×			×			×		
PreAlb		×								×							×
Outcomes		×															×
Concomitant medication		×	×	×	×	×	×	×	×	×	×	×	×	×	×	×	
Adverse events		×	×	×	×	×	×	×	×	×	×	×	×	×	×	×	
Mortality rate																	After 28 days

*Abbreviations*: *ALT* alanine aminotransferase, *AST* aspartate aminotransferase, *APACHE II* Acute Physiology and Chronic Health Evaluation II, *Cl* chloride, *Cr* creatinine, *GCS* Glasgow coma scale, *GRV* gastric residual volume, *K* potassium, *Mg* magnesium, *Na* sodium, *NUTRIC* Nutrition Risk in Critically Ill, *P* phosphorus, PreAlb pre-albumin, *SOFA* sequential organ failure assessment, *TG* triglyceride

### Description of evaluations

As shown in Fig. 2, calorie achievement goals are set according to the patients’ modified NUTRIC score. In everyday visits, we will evaluate GI signs and symptoms (e.g. vomiting, diarrhea, abdominal distention) and gastric residual volume (GRV). If there is no sign or symptom of intolerance and GRV is less than 250 ml, EN will be increased by 10%. Otherwise, we will approach as shown in Fig. 3. Energy homeostasis (calorie intake-estimated calorie requirement) will be recorded each day. Mid-arm circumference, which is an available anthropometric measurement tool, will be evaluated twice a week. As all patients receiving EN should be monitored for some clinical and laboratory variables, we set our monitoring approach as reported by Berger MM, et al. [34], Monitoring nutrition in the ICU, Clinical Nutrition (2018). Concomitant medications, pressure ulcers, infectious complications, and other adverse events will be recorded every day. The APACHE II and Sequential Organ Failure Assessment (SOFA) will be scored on days 0, 8, and 15. Before and after the intervention, fasting blood and 24-h urine samples will be obtained. Urine urea nitrogen, 3-Methyl histidine, and creatinine will be measured in urine samples. Fasting blood glucose, insulin, C-reactive protein (CRP) and endotoxins will be measured in fasting blood samples.

## Safety assessment

Despite the ample evidence supporting the safety of probiotics in critically ill patients, there are case reports of risks and suggested theoretical risks related to probiotic administration. The most important is the risk of bacteremia and fungemia in high-risk populations, which may be associated with improper use and unintended contamination of central-line catheters [33]. To avoid risk of bacteremia we will not include a high-risk population, such as patients who have recently had major surgery, or patients with short bowel syndrome, heart valve disease, artificial heart valve, or patients who are immunocompromised. We will also pay careful attention to the proper administration and handwashing protocols. Gene transfer and over-stimulation of the immune system are other suggested theoretical risks, on which there is not yet any evidence in humans [33].

## Intervention discontinuation

If intervention-related side effects exceed the level reported by previous studies, we will stop the intervention and present the results to the Ethics Committee of Mashhad University of Medical Sciences (MUMS) for further decision making. If adverse events are caused by the study intervention, the researcher and medical team will provide timely and proper treatment to participants.

## Statistical considerations

### General design issues

Data will be analyzed using the intention-to-treat approach.

### Sample size and randomization

We did not find any similar study that has evaluated our primary objectives. So, we considered one of the main secondary objectives to estimate the required sample size. Mahmoodpoor and co-workers [35] reported the ICU stay in two study groups as 18.6 ± 8 and 11.6 ± 6.3 days. Considering alpha error of 0.05 and power of 80%, the required sample size allowing for 10% dropout is 20 patients in each group.

### Outcomes

#### Primary outcomes

The primary outcomes are

1. Energy homeostasis (calorie intake-estimated calorie requirement)
2. Protein catabolism (nitrogen balance)

- Nitrogen balance is a measure of the net change in total body protein. It is the difference between nitrogen eliminated from the body and nitrogen ingested in the diet. A positive or neutral nitrogen balance shows that protein stores are increased or maintained, while a negative nitrogen balance indicates protein mass is decreasing. The practical method for estimating nitrogen balance supposes that total nitrogen loss is equal to urinary urea nitrogen excretion plus 4 g/day additional loss from non-urinary urea nitrogen, gastrointestinal, and insensible losses [36, 37]. To measure the nitrogen balance, during the 24-h urine collection, the total intake of protein will be recorded to calculate nitrogen intake: 24-h urine samples will be immediately delivered to the laboratory to measure urea nitrogen.

3. Muscle protein degradation (3-methyl histidine (3MH) in 24-h urine)

- 3MH is exclusively found in muscle proteins, and after protein degradation, it is rapidly excreted in the urine without further reutilization or metabolization. So, measuring urinary 3MH, after at least 1 day of a meat-free diet, can be used as a biomarker of muscle protein breakdown [38, 39]. After a 1-day meat-free diet, 24-h urine will be collected. Urine samples will be centrifuged for 20 min at 1000×g. The supernatant will be collected and stored at − 70 °C for a maximum of 2 months. The ELISA method will be used for 3MH detection.

4. Muscle protein turnover (3MH/creatinine ratio in 24-h urine)

- Since 24-h urinary creatinine estimates the total pool of muscle proteins, muscle protein turnover can be calculated from the 3MH/creatinine excretion ratio [38]. A 24-h urine sample will be delivered to the laboratory to immediately measure creatinine by the enzymatic method.

5. Lipolysis (free glycerol in serum)

- Free glycerol is an important index of lipid metabolism. When the body uses stored fat as the energy supply, glycerol and fatty acids are released into the circulation. The absence of glycerol kinase in the adipocyte decreases triacylglycerol resynthesize and supports hepatic gluconeogenesis [40]. After obtaining the overnight fasting blood sample, the serum will be separated. The serum sample will be stored at − 70 °C for further measurement of free glycerol by enzymatic colorimetric method.

6. Glucose homeostasis (fasting blood sugar (FBS), insulin)
7. Inflammatory status (CRP, neutrophil/lymphocyte ratio (NLR))

- NLR is an available measurable marker used to measure systemic inflammation.

8. Dysbiosis status and luminal integrity (endotoxin levels)

- Intestinal gram-negative bacteria are the major source of lipopolysaccharides (LPS), which are referred to as endotoxins. In the case of reduced intestinal barrier integrity due to dysbiosis, luminal endotoxins can enter the circulation [41]. Endotoxin activity assay (EAA) will be used to determine endotoxin levels in whole blood.

#### Secondary outcomes

The secondary outcomes are:

1. Enteral feeding tolerance (abdominal examination and GRV measurement)
2. Clinical prognosis (APACHE and SOFA score)
3. Nutritional status (NUTRIC score)
4. Infectious complications incidence
5. Pressure ulcer incidence and grade
6. Ventilator-dependent days
7. Length of ICU stay
8. Length of hospital stay
9. 28-Day mortality

### Data analysis

Data will be analyzed using SPSS for windows version 11.5 and MedCalc Statistical Software version 18.11.3 (MedCalc Software bvba, Ostend, Belgium; https://www.medcalc.org; 2019). Descriptive (frequency, percentage, mean, standard deviation) and inferential analysis (student *t* test, paired sample *t* test, repeated measures analysis of variance (ANOVA)) will be performed. Any covariates will be controlled by ANCOVA or binary logistic regression. All tests will be two-tailed and a *p* value <0.05 will be considered as statistically significant.

## Data collection and quality assurance

Data collection will be supervised by the primary investigator. In addition, 10% of electronic data will be checked randomly with paper questionnaires and any discrepancies will lead to a 50% double-checking of electronic data. Any outliers will be checked against patient medical records.

## Discussion

In the intestinal tract, gut microbiota control different immune and endocrine functions [42]. They have a major role in the absorption, storage, and consumption of energy derived from the diet [14, 15]. Outside the intestine, they also modulate cell metabolism, energy homeostasis, systemic inflammation, appetite and food intake [42, 43] . On the other hand, a few clinical studies, modulating gut microbiota in patients under critical care demonstrated a faster return of gut function and earlier achievement of the nutritional target dose [16, 17]. Therefore, we expect that our patients in the intervention group will have a better enteric feeding tolerance and also more desirable energy homeostasis.

Animal studies have shown that modulation of gut microbiota by prebiotics, probiotics, or synbiotics can reduce cachexia and muscle mass sparing [22–25]. The underlying mechanisms remain to be identified. Gut microbiota influence amino acid bioavailability and are a source of different metabolites such as conjugated linoleic acid, acetate, and bile acids that modulate muscle metabolism. Gut microbiota are also a source of PAMPs, which activate the transcription factor NF-kB through toll-like receptors (TLRs) and cause muscle wasting. Gut microbiota also modulate production of proinflammatory cytokines, which can induce muscle atrophy [8]. We expect that synbiotic intervention in patients under critical care reduces muscle protein degradation and turnover. As malnutrition and muscle wasting in these patients are associated with negative health outcomes, gut microbiota modulation will improve the clinical prognosis and reduce infectious complications, ventilator dependency, and length of ICU stay.

## Trial status

Recruitment was started on 1 March 2019 and is estimated to be completed by October 2019. Recruitment was ongoing at the time of submission. This is the last protocol version (Number 5, 15 January 2020).

## Additional file

**Additional file 1.** Standard protocol items: recommendation for interventional trials (SPIRIT) 2013 Checklist: Recommended items to address in a clinical trial protocol and related documents.

## Data Availability

The datasets generated and/or analyzed during the current study are not publicly available due to ethical considerations, but may be available from the corresponding author on reasonable request.

## Abbreviations

3MH: 3-Methy histidine
AIDS: Acquired immune deficiency syndrome
ALT: Alanine aminotransferase
ANCOVA: Analysis of covariance
ANOVA: Analysis of variance
APACHE II: Acute Physiology and Chronic Health Evaluation II
AST: Aspartate aminotransferase
CFU: Colony-forming units
Cl: Chloride
Cr: Creatinine
CRP: C-reactive protein
EAA: Endotoxin activity assay
ELISA: Enzyme-linked immunosorbent assay
EN: Enteral nutrition
ESPEN: European Society of Parenteral and Enteral Nutrition
GCS: Glasgow coma scale
GIT: Gastrointestinal tract
GRV: Gastric residual volume
ICU: Intensive care unit
K: Potassium
LPS: Lipopolysaccharides
Mg: Magnesium
MOF: Multi-organ failure
Na: Sodium
NF-kB: Nuclear factor kappa light chain enhancer of activated B cells
NGT: Nasogastric tube
NLR: Neutrophil\lymphocyte ratio
NUTRIC: Nutrition risk in critically ill
P: Phosphorus
PAMPs: Pathogen-associated molecular patterns
PreAlb: Pre-albumin
SOFA: Sequential Organ Failure Assessment
TG: Triglyceride
TLRs: Toll-like receptors

## Footnotes

1 Acute Physiology and Chronic Health Evaluation II

2 Nutrition Risk In Critically Ill
